# Depression in Childhood Asthma vs. Adult-Onset Asthma: A Cross-Sectional Study from the National Health and Nutrition Examination Survey (NHANES)

**DOI:** 10.3390/children9121797

**Published:** 2022-11-23

**Authors:** Zeeshan Faruqui, Zalak Thakker, Dilshad Parveen, Saloni Naik, Marzhan Urazbayeva, Vidisha Jain, Dhivya Kannan, Che Marie, Sona Xavier, Patali Mandava, Joshua Jogie, Garima Yadav, Saral Desai, Ya-Ching Hsieh, Urvish Patel, Devraj Chavda, Jagdeep Kaur

**Affiliations:** 1 Department of Psychiatry, Keystone Health Center, Chambersburg, PA 17201, USA; 2 Department of Pediatrics, Bassett Healthcare Network, 1 Atwell Road, Cooperstown, NY 13326, USA; 3 Department of Pediatrics, Ganesh Shankar Vidyarthi Memorial Medical College, Kanpur 208002, India; 4 Department of Psychiatry, Pramukhswami Medical College, Karamsad, Anand 388345, India; 5 GI Medicine & Pediatrics, Baylor College of Medicine, Houston, TX 77030, USA; 6 Department of Pediatrics, Jawaharlal Nehru Medical College, Ajmer 305001, India; 7 Department of Pediatrics, Government Villupuram Medical College and Hospital, Villupuram 605601, India; 8 Neonatal Special Care Unit, Port of Spain General Hospital, Port-of-Spain 150123, Trinidad and Tobago; 9 Behavioral Medicine and Psychiatry, Jackson Park Hospital, 7531 S Stony Island Ave, Chicago, IL 60649, USA; 10 Department of Medicine, Diabetes Care Institute, University of Washington, Seattle, WA 98109, USA; 11 Department of Neonatology, Port-of-Spain General Hospital, Port-of-Spain 150123, Trinidad and Tobago; 12 Department of Psychiatry, Bronx Care Health System, Bronx, NY 10456, USA; 13 Department of Psychiatry, Tower Health—Phoenixville Hospital, Phoenixville, PA 19460, USA; 14 Department of Public Health, Icahn School of Medicine at Mount Sinai, New York, NY 10029, USA; 15 Department of Neurology, Icahn School of Medicine at Mount Sinai, New York, NY 10029, USA; 16 Department of Pediatrics-Neurology, Sunrise Children’s Hospital, Las Vegas, NV 89109, USA; 17 Department of Psychiatry and Behavioral Sciences, University of California Davis, Sacramento, CA 95817, USA

**Keywords:** asthma, depression, childhood, adulthood, substance use, predictors, public health, pediatrics, psychiatry

## Abstract

Background: asthma, a chronic respiratory disease caused by inflammation and narrowing of the small airways in the lungs, is the most common chronic childhood disease. Prevalence of childhood asthma in the United States is 5.8%. In boys, prevalence is 5.7% and it is 6% in girls. Asthma is associated with other comorbidities such as major depressive disorder and anxiety disorder. This study explores the association between asthma and depression. Methods: we conducted a retrospective cross-sectional study using NHANES data from 2013 to 2018. Asthma and childhood onset asthma were assessed using questionnaires MCQ010 and MCQ025, respectively. Sociodemographic variables were summarized, and univariate analysis was performed to determine the association between asthma and major depressive disorder and its individual symptoms. Results: there were 402,167 participants from 2013–2018 in our study: no asthma in 84.70%; asthma in 15.30%. Childhood onset asthma (COA) included 10.51% and adult-onset asthma (AOA) included 4.79%. Median age of COA is 5 years and AOA is 41 years. Among the asthma groups, most AOA were females (67.77%, *p* < 0.0001), most COA were males (52.16%, *p* < 0.0001), and ethnicity was predominantly White in AOA (42.39%, *p* < 0001) and in COA (35.24%, *p* < 0.0001). AOA mostly had annual household income from $0–24,999 (35.91%, *p* < 0.0001), while COA mostly had annual household income from $25,000–64,999 (36.66%, *p* < 0.0001). There was a significantly higher prevalence of MDD in COA (38.90%) and AOA (47.30%) compared to NOA (31.91%). Frequency of symptoms related to MDD were found to have a significantly higher prevalence and severity in the asthma groups compared to no asthma, and slightly greater and more severe in AOA than in COA. Symptoms include having little interest in doing things (COA 18.38% vs. AOA 22.50% vs. NOA 15.44%), feeling down, depressed, or hopeless (COA 20.05% vs. AOA 22.77% vs. NOA 15.85%), having trouble sleeping or sleeping too much (COA 27.38% vs. AOA 23.15% vs. NOA 22.24%), feeling tired or having little energy (COA 39.17% vs. AOA 34.24% vs. NOA 33.97%), having poor appetite or overeating (COA 19.88% vs. AOA 20.02% vs. NOA 15.11%), feeling bad about yourself (COA 13.90% vs. AOA 13.79% vs. NOA 10.78%), having trouble concentrating on things (COA 12.34% vs. AOA 14.41% vs. NOA 10.06%), moving or speaking slowly or too fast (COA 8.59% vs. AOA 9.72% vs. NOA 6.09%), thinking you would be better off dead (COA 3.12% vs. AOA 4.38% vs. NOA 1.95%) and having the difficulties these problems have caused (COA 21.66% vs. AOA 26.73% vs. NOA 19.34%, *p* < 0.0001). Conclusion: MDD and related symptoms were significantly higher and more severe in participants with asthma compared to no asthma. Between adult-onset asthma compared to childhood onset asthma, adult-onset asthma had slightly greater and more severe MDD and related symptoms compared to childhood onset asthma.

## 1. Introduction

Asthma is a chronic respiratory disease caused by inflammation and narrowing of the small airways in the lungs [1]. It is the most common chronic childhood disease [1]. The current prevalence of childhood asthma in the United States is 5.8%, representing a prevalence rate of 5.7% in boys and 6% in girls [2]. Black children (12.3%) are likely to have asthma more than two times as compared to White children (5.5%) [2]. Childhood asthma is associated with many comorbidities such as major depressive disorder (MDD) and anxiety disorder [3,4].

A study based on National Health and Nutrition Examination Survey (NHANES) data observed that depression was present in more than one-third of older adults with asthma [5]. Together, they led to poor asthma outcomes including increased ED/urgent care visits [5]. A more recent cross-sectional study found higher rates of combined anxiety and depression than isolated anxiety and depression in children with asthma. Moreover, higher numbers of combined anxiety and depression were observed in children with uncontrolled asthma [6]. Letitre concluded that increased risk of depression, anxiety and poor self-esteem were not found in children with persistent well-controlled asthma in a small cross-sectional study [7]. Groot found asthma to be linked with various comorbidities, including childhood depressive illnesses, though there was poor evidence of the impact of comorbidities on asthma [8].

Although studies have shown the association of depression and asthma, there were only a few studying the prevalence of depression in childhood asthmatics in the last decade [8]. The existing literature either has a small study sample size or has a cohort that cannot be generalized to the population of the USA [6,7]. Moreover, studies with a large sample size are limited to older adults [5,9]. Therefore, in this study, we aim to explore the relationship that exists between childhood asthma and the prevalence of depression.

## 2. Materials and Methods

### 2.1. Details of the Data

NHANES is a nationally representative survey-based program maintained by the Centers of Disease Control and Prevention, National Center for Health Statistics (NCHS). It is a cross-sectional survey that aims to evaluate the health and nutritional condition of the US population spread across the country. A sample is selected following a multistage probability sampling design for a 2-year cycle, which is representative of the civilian non-institutionalized US population. The survey includes interviews regarding demographics, socioeconomic, dietary and health-related questions followed by a physical examination and sample collection by trained medical professionals. Information on methods and sampling are provided on the CDC website: https://wwwn.cdc.gov/nchs/nhanes/ (accessed on 11 April 2022).

### 2.2. Study Population

We conducted a population-based, retrospective cross-sectional study using the NHANES database from 2013–2018. We downloaded the dataset from the NHANES website that is sponsored by the CDC and is freely and publicly available. IRB and informed consent were not required for this study. 

There were three groups in our study: (a) childhood onset asthma (COA), (b) adult-onset asthma (AOA) and (c) no asthma (NOA). All the participants giving positive responses for the following two questions were defined as current asthma: “Has a doctor or other health professional ever told (you/SP) that (you/have/s/he/SP has) asthma?” (MCQ010) and “During the past 12 months, (have you/has SP) had an episode of asthma or an asthma attack?” (MCQ040). Childhood-onset asthma was defined by asking the question: “How old (were you/was SP) when (you were/s/he was) first told (you/he/she) had asthma (az-ma)?” (MCQ025). We included sociodemographic variables such as age, gender, race and median household income, comorbidities such as hypertension, dyslipidemia, depression, obesity, trouble sleeping and diabetes. Participants with missing data on demographics were excluded from this study.

### 2.3. Measures

The primary measure of the research included symptoms of major depressive disorder. For secondary measures, we looked at predictors of major depressive disorder and substance use in both childhood- and adult-onset asthma. 

#### 2.3.1. Acute Exacerbation of Asthma

The recurrent emergency hospital visits were assessed by the following question: “[During the past 12 months], {have you/has SP} had to visit an emergency room or urgent care center because of asthma (az-ma)?” (MCQ050).

#### 2.3.2. Depression

The primary outcome of our study is depression and it is assessed by the responses from the mental health depression screener (DPQ_l) questionnaires. The components consist of a nine-item depression screening instrument with variable name prefix DPQ. The list includes questions such as the following: “Over the last two weeks, how often have you been bothered by feeling tired or having little energy?” (DPQ040). “Over the last two weeks, how often have you been having little interest or pleasure in doing things?” (DPQ010). “Over the last two weeks, how often have you been having trouble falling or staying asleep or sleeping too much?” (DPQ030).

#### 2.3.3. Comorbid Conditions

The sleep disturbances of the individuals were assessed by the following questions: “{Have you/Has SP} ever told a doctor or other health professional that {you have/s/he has} trouble sleeping?” (SLQ050). “How much sleep {do you/does SP} usually get at night on weekdays or workdays?” (SLD010H). The patients’ weight histories were assessed by the following questions: “How much {do you/does SP} weigh without clothes or shoes?” (WHD020) “These next questions ask about {your/SP’s} height and weight at different times in {your/his/her} life. How tall {are you/is SP} without shoes? (WHD010) How old {was SP/were you} when a doctor or other health professional first told {you/him/her} that {you/he/she} had diabetes or sugar diabetes? (DID040) During the past 12 months, how many times has a doctor or other health professional checked {you/SP} for glycosylated hemoglobin or ‘A one C’?” (DID270).

#### 2.3.4. Substance Use

Substance use, such as of cocaine, heroin, methamphetamine, marijuana, injectables, etc., was assessed using the following questions: DUQ200, DUQ100, DUQ250, DUQ290, DUQ330 and DUQ370.

### 2.4. Statistical Analysis

All statistical analysis was completed using Statistical Analysis System (SAS) software, version 9.4 or later (SAS Institute, Cary, NC, USA). All sample data were weighted before analysis, as is done with NHANES data. We performed univariate analysis to find association of e-cigarettes with cardiovascular disease and other sociodemographic variables using chi square for categorical variables and paired/unpaired t-test/Wilcoxon rank sum test/Mann–Whitney test for continuous variables. Multivariable logistic regression models were generated to predict the association of depression and asthma adjusting for confounding variables to estimate odds ratio (OR) and 95% confidence intervals. The *p* value of <0.05 is considered statistically significant. 

## 3. Results

There were a total of 402,167 participants from 2013–2018 in our study. Participants with no asthma were 340,647 (84.70%) and with asthma were 61,520 (15.30%). Childhood-onset asthma (COA) included 42,275 (10.51%) and adult-onset asthma (AOA) included 19,245 (4.79%). COA was diagnosed at the median age of 5 years and adult onset at 41 years. COA was more prevalent in males in comparison with females (11.33% vs. 9.75%, *p* < 0.0001) and AOA was more prevalent in females in comparison with males (6.29% vs. 3.19%, *p* < 0.0001) [Table 1]. 

COA was most prevalent in “other” race, followed by non-Hispanic Black, other Hispanics, non-Hispanic White and Mexican American (14.56% vs. 14.04% vs. 10.64% vs. 10.15% vs. 7.93%, *p* < 0.0001), while AOA was most common in non-Hispanic White, followed by other Hispanic, non-Hispanic Black, other race, and Mexican American (5.56% vs. 5.09% vs. 4.94% vs. 4.73% vs. 3.85, *p* < 0.0001) [Table 1].

Hypertension was the most common comorbidity among all three groups, the highest percentage being in AOA then in no asthma group and COA group (86.12% vs. 80.31% vs. 78.64%, *p* < 0.0001). The AOA group had a higher median BMI (30.3, *p* < 0.0001) than the no asthma group (25.6, *p* < 0.0001) and COA group (24.7, *p* < 0.0001). In participants who had comorbid trouble sleeping, AOA group had the highest percentage (49.08%, *p* < 0.0001) in comparison to COA group (34.10%, *p* < 0.0001) and no asthma group (24.53%, *p* < 0.0001). Similarly, the AOA group had a higher percentage with high cholesterol (49.32%, *p* < 0.0001) than the no asthma group (34.64%, *p* < 0.0001) followed by the COA group (23.43%, *p* < 0.0001). Diabetes mellitus was highest in AOA then no asthma group and COA (25.05%, 9.58%, 5.97%, *p* < 0.0001). AOA had higher prevalence of comorbidities including COPD, chronic bronchitis and emphysema (16.36%, 25.25%, 8.07%, *p* < 0.001), followed by COA (7.52%, 14.98%, 3.67%, *p* < 0.0001) and no asthma group (2.61%, 3.53%, 1.18%, *p* < 0.0001). E-cigarette use (32.09% vs. 17.31%), smoking (21.41% vs. 18.12%), high alcohol intake (50.98% vs. 44.02%), cocaine abuse (97.14% vs. 93.53%), marijuana abuse (62.22% vs. 55.42%) and heroin abuse (18.89% vs. 18.74%) were more prevalent in COA in comparison with AOA while methamphetamine abuse (41.99% vs. 42.03%) was less common in COA in comparison with AOA [Table 1].

The prevalence of MDD symptoms were much higher in AOA group compared to COA group and NOA group (47.30% vs. 38.90% vs. 31.91%, *p* < 0.0001). The NOA group compared to the COA and AOA groups had the highest percentage (76.14% vs. 71.04% vs. 62.45%, *p* < 0.0001) to answering “NOT AT ALL” to the question: “Have little interest in doing things.” The COA group has the highest percentage compared to the AOA and NOA groups (6.40% vs. 6.27% vs. 4.68%, *p* < 0.0001) to answering, “More than half the days” to the question: “Have little interest in doing things.” The AOA group had the highest percentage compared to COA and NOA (16.07% vs. 10.26% vs. 7.17%, *p* < 0.0001) to answering, “Nearly every day” to the question: “Trouble sleeping or sleeping too much.” For the question, “Feeling tired or having little energy”, the COA group had the highest percentage compared to the AOA and NOA (39.17% vs. 34.24% vs. 33.97%, *p* < 0.001) groups by answering, “Several days”. For questions like “Poor appetite or overeating”, “Trouble concentrating on things”, “Moving or speaking slowly or too fast” and “Difficulty these problems have caused”, the AOA have the highest percentage (20.02% vs. 14.41% vs. 9.72 % vs. 26.73%) as they answered “Several days” compared to the COA and AOA groups [Table 2].

### Multivariate Regression Analysis

In regression analysis, COA was associated with higher prevalence odds of high alcohol intake (aOR 1.06, 95%CI 1.06–1.06, *p* < 0.0001), smoking (1.19, 1.19–1.19, *p* < 0.00010, cocaine use (67.99, 67.67–68.31, *p* < 0.0001), methamphetamine use (1.67, 1.67–1.67, *p* < 0.0001) and non-Hispanic Black race (1.04, 1.04–1.04, *p* < 0.0001). AOA was associated with 164% higher odds of major depression disorder [2.64, 2.64–2.64, *p* < 0.0001] and higher prevalence odds of marijuana use (2.53, 2.53–2.54, *p* < 0.0001), heroin use (1.82, 1.82–1.83, *p* < 0.0001), and non-Hispanic Asian race (2.24, 2.24–2.25, *p* < 0.0001) [Table 3].

## 4. Discussion

### 4.1. Epidemiology and Demographics

Our retrospective cross-sectional study included individuals with AOA, COA and NOA from the NHANES dataset (2013–2018) representing the US population. This study assessed the prevalence of COA and AOA, associated comorbidities, and prevalence of MDD and related symptoms in COA vs. AOA vs. NOA. We found the prevalence of COA was nearly two times more compared to AOA, which is inconsistent with the current national prevalence. 

A study done by Cynthia et al. in the US population from 2006–2018 demonstrated only slightly higher prevalence of COA compared to AOA (8.1% vs. 7.9%) [10]. This difference could be because we included individuals only from the 2013–2018 NHANES dataset. COA was diagnosed at the median age of 5 years, which was the same as a study done by Lakshminarasappa [6], and AOA was diagnosed at the median age of 41 years, similar to studies done by Mirabelli [11] and Ilmarinem [12] in which the median age at onset was 38 years and 46 years, respectively. Our results are consistent with the literature that COA is more prevalent in males, while AOA is more prevalent in females [13,14,15,16,17]. This gender-related switch has been explained by many hypothesis like smaller airways in boys compared to girls [18] and increase in the activities of sex hormones in females starting from puberty [19,20]. AOA, as well as COA, is more prevalent among Whites in our study, which appears to be inconsistent with national data and other literature [1,21,22]. Similarly, another national-level study conducted by Keet et al. concluded that the Black race and Puerto Rican ethnicity was a risk factor in developing asthma in children and a study done by Holt et al. revealed that COA was higher among children of mothers who were Puerto Rican and Black than White (40.4% vs. 23.7% vs. 12.6%) [23,24]. Likewise, Piccirillo’s study done on the US Armed Forces concluded that the Black ethnicity enrolled in the army had a higher disability evaluation for asthma compared to the Whites. Additionally, the race designated as “Other” had higher rates when compared to the Whites in the Marine Corps and the Army [25]. Low and median annual household income groups had more prevalence of asthma compared to higher income groups, which is similar to the national data and that of the American Lung Association [1,26]. Higher prevalence in low to median income groups can be due to poor housing quality and social inequalities [27,28]. 

### 4.2. Comorbidities

The results indicated that hypertension was the most common comorbidity among all three groups, with the highest percentage in AOA. Similar results were obtained by Tsai in their study, which found comorbid hypertension higher in percentage in adult asthmatics >55 years of age [29]. Christiansen found hypertension to display augmented asthma morbidity [30]. High alcohol intake was the second most common comorbidity with the highest percentage in AOA. Patra also observed that drinking independently affected wheezing symptoms and diagnosed asthma [31]. Contrary to our study, Leiberoth discovered the risk of asthma in heavy drinkers to be statistically insignificant. The study also found moderate drinking to be protective against developing asthma [32]. Higher median BMI was found in the AOA group compared to COA and no asthma. Obesity contributes to low-grade systemic inflammation, which can lead to airway inflammation and asthma exacerbation [33]. This can be mediated by adipokines including IL-6, TNF alpha, leptin and adiponectin [34]. Mechanical factors in obesity can contribute to low-volume breathing, which increases airway responsiveness and reduces response to medication [35,36]. The AOA group also had a higher percentage of high cholesterol, which is consistent with the findings of Ramaraju. Their study found a positive association between serum cholesterol and asthma. Hypercholesterolemia enhances the proinflammatory phenotype in microcirculation [37] and increases pulmonary allergic inflammation [38]. E-cigarette use was the only comorbidity with the highest percentage in COA. This can be associated with increased exposure to e-cigarettes in children and adolescents [39]. In addition, e-cigarettes possess a respiratory threat, more in adolescents with asthma [40].

### 4.3. Asthma and Major Depressive Disorder

Many times, asthmatic patients present with depression as a complication of their long-term illness, which affects their disease control and quality of life. In a study done by Wufuer, he studied the incidence of depression and its influencing factors in 387 patients with asthma [41]. Logistic regression analysis indicated that the severity of asthma symptoms, taking asthma medication, the frequency of asthma attacks, lack of education and Uighur ethnicity were the major risk factors for depression in this population of patients with asthma [41]. We also found in our study that asthmatic patients presented with symptoms of depression more as compared with non-asthmatics.

According to the study conducted by Haley Morin [42], patients who had both depression and anxiety symptoms during hospitalization were most likely to have persistent symptoms at follow-up after their discharge; therefore, screening at both time points during and after hospitalization may be useful to identify mental health symptoms. In his study, 53% had elevated symptoms of depression, anxiety or both either during hospitalization or after discharge. During hospitalization, 38% had elevated depression symptoms and 45% had elevated anxiety symptoms [42]. Among 96 participants who completed the study who were at post-discharge follow-up, 18% had elevated depression symptoms and 20% had elevated anxiety symptoms. The prevalence of MDD symptoms were much higher in the AOA group compared to the COA group and NOA group in our study.

In the study done by Jiang M [43], he found that varying levels of cytokines play an important role in the presentation of both asthma and depression. He recorded that the biological connection of the two illnesses was significant, and cytokines were increased in the depressed as compared to the non-depressed people [43]. In people with depression, concentration of monocytes related to cytokines such as IL-1 was significantly higher than that in non-depressive control subjects. 

According to Liu et al., major depression and asthma frequently co-occur, suggesting a shared genetic vulnerability between these two disorders [44]. David S Bennett [45] did a meta-analysis study where he found that depressive symptoms among children with chronic medical problems were more commonly seen as compared with healthy children [45]. A great variability in depressive symptoms was found across children with the same disorder; children with certain disorders such as asthma and sickle cell anemia may be at greater risk than children with other disorders such as cancer and cystic fibrosis. According to the study done by Rajhans P et al. [46], they found that overall prevalence of psychiatry morbidity was 33.3%, and they also identified other additional problems such as specific phobia, conduct disorder and ADHD, but depression was the most prevalent [46].

The child being diagnosed with asthma itself puts the children under stress, as there is no permanent cure and it requires lifelong use of medications and lifestyle changes. This may lead to fear and stress, finally leading to anxiety and depression. In addition, anxiety and depression is also seen in greater frequency in caregivers of asthmatic patients, which may further influence their anxiety, therefore leading to a vicious cycle. In a study done by Easter G [47], he found the prevalence of depressive (44%) and anxiety (50%) symptoms were higher in caregivers of children with asthma as compared to controls [47]. The psychiatric comorbidities of caregivers have a negative impact on childhood asthma, increasing the severity, with poor asthma control and an increase in the medication use.

### 4.4. Strengths and Limitations

There are some limitations in this study. NHANES is a self-reported cross-sectional survey and study, so this survey is likely to be affected by recall bias and causal or temporal association could not be established. The diagnosis of asthma and depression was based on self-reports without clinical validation, which may result in recall bias. Since our study compared between two categories like childhood- and adult-onset asthma, it is a challenge looking for the literature as there are wide differences in the literature and we acknowledge this limitation. It is difficult to accurately compare and contrast childhood- and adult-onset asthma because of existing gaps in the literature and we acknowledge this limitation. However, there were strengths in the study as well. The study is of a large and nationwide dataset across the US. The concept of comparing childhood- and adult-onset asthma is a novel one that not many studies have done.

## 5. Conclusions

MDD and related symptoms were found to be more common and severe in asthmatic patients compared to no asthma. Between asthma groups, adult-onset asthma had slightly greater and more severe MDD and related symptoms compared to childhood-onset asthma. Given the high rate of co-occurrence of asthma and MDD, primary care providers and asthma specialists should routinely screen all asthmatic patients for symptoms of MDD and refer the patients showing depressive symptoms to mental health services to help improve the compliance in asthma care.

## Figures and Tables

**Table 1:** Demographic and comorbidity characteristics of asthma amongst US population (2013–2018).

Variables	Childhood Asthma *n* = 42,275 (10.51%)	Adult Asthma *n* = 19,245 (4.79%)	No Asthma *n* = 340,647 (84.70%)	Total *n* = 402,167 (100%)	*p*-Value
Demographic and Socioeconomic Characteristics (%)					
Age					
Age (median (Q1–Q3))	5 (2–9)	41 (30–55)	-	-	<0.0001
Gender					
Male	22,051 (52.16)	6202 (32.23)	166,432 (48.86)	194,685 (48.41)	<0.0001
Female	20,224 (47.84)	13,043 (67.77)	174,215 (51.14)	207,482 (51.59)	<0.0001
Race					
Mexican American	5185 (12.26)	2518 (13.08)	57,641 (16.92)	65,344 (16.25)	<0.0001
Other Hispanic	4440 (10.50)	2126 (11.05)	35,163 (10.31)	41,729 (10.38)	<0.0001
Non-Hispanic White	14,896 (35.24)	8158 (42.39)	123,686 (36.31)	146,740 (36.49)	<0.0001
Non-Hispanic Black	11,571 (27.37)	4072 (21.16)	66,767 (19.60)	82,410 (20.49)	<0.0001
Non-Hispanic Asian	3168 (7.49)	1392 (7.23)	40,677 (11.94)	45,237 (11.25)	<0.0001
Other Race	3015 (7.13)	979 (5.09)	16,713 (4.91)	20,707 (5.15)	<0.0001
Annual household income					
$0–24,999	11,476 (28.81)	6405 (35.91)	84,582 (26.90)	102,463 (27.54)	<0.0001
$25,000–64,999	14,600 (36.66)	6043 (33.88)	11,3821 (36.20)	134,464 (36.14)	<0.0001
$65,000–99,999	5939 (14.91)	2288 (12.83)	49,174 (15.64)	57,401 (15.43)	<0.0001
$100,000 and more	7812 (19.61)	3101 (17.39)	66,854 (21.28)	77,767 (20.90)	<0.0001
Comorbidities (%)					
COPD	1575 (7.52)	3097 (16.36)	5861 (2.61)	10,533 (3.98)	<0.0001
Chronic Bronchitis	3137 (14.98)	4780 (25.25)	7947 (3.53)	15,864 (5.99)	<0.0001
Emphysema	768 (3.67)	1527 (8.07)	2657 (1.18)	4952 (1.87)	<0.0001
Congestive Heart Failure	779 (3.72)	1766 (9.33)	6880 (3.06)	9425 (3.56)	<0.0001
Coronary Heart Disease	878 (4.19)	1589 (8.40)	9889 (4.40)	12356 (4.67)	<0.0001
Hypertension	5787 (78.64)	9364 (86.12)	67,682 (79.71)	82,833 (80.31)	<0.0001
High Cholesterol	5972 (23.43)	9421 (49.32)	84,393 (34.64)	99,786 (34.62)	<0.0001
BMI (median(Q1–Q3)	24.7 (19.7–30.7)	30.3 (25.8–36.1)	25.6 (20.5–30.5)	-	<0.0001
Diabetes Mellitus	2525 (5.97)	4821 (25.05)	32,631 (9.58)	39,977 (9.94)	<0.0001
Stroke	761 (3.63)	1790 (9.46)	7871 (3.50)	10,422 (3.94)	<0.0001
Cancer/Malignancy	2155 (10.29)	2914 (15.40)	24,096 (10.71)	29,165 (11.01)	<0.0001
Trouble Sleeping	8693 (34.10)	9376 (49.08)	59,766 (24.53)	77,835 (27.01)	<0.0001
Liver Conditions	1095 (5.23)	1663 (8.79)	10,276 (4.57)	13,034 (4.92)	<0.0001
High Alcohol	8552 (50.98)	4777 (44.02)	70,096 (47.33)	83,425 (47.48)	<0.0001
E-Cigarette	4818 (32.09)	2076 (17.31)	24,295 (16.07)	31,189 (17.51)	<0.0001
Smoking	4961 (21.41)	3440 (18.12)	37,547 (16.09)	45,948 (16.68)	<0.0001
Cocaine	3734 (97.14)	2401 (93.53)	26,964 (95.30)	33,099 (95.38)	<0.0001
Marijuana	11,190 (62.22)	4956 (55.42)	71,389 (51.67)	87,535 (53.02)	<0.0001
Methamphetamine	1614 (41.99)	1079 (42.03)	11,130 (39.34)	13,823 (39.83)	<0.0001
Heroin	726 (18.89)	481 (18.74)	4102 (14.50)	5309 (15.30)	<0.0001

**Table 2:** Univariate analysis of childhood and adult responses to major depressive disorder and low-mood-related questions.

Variables	Childhood Asthma *n* = (%)	Adult Asthma *n* = (%)	No Asthma *n* = (%)	Total *n* = (%)	*p*-Value
Prevalence (%)					
Major Depressive Disorder (%)					
No	13,566 (61.10)	9356 (52.70)	148,807 (68.09)	171,729 (66.43)	<0.0001
Yes	8637 (38.90)	8398 (47.30)	69,740 (31.91)	86,775 (33.57)	<0.0001
Have little interest in doing things (%)					
Not at all	15,773 (71.04)	11,807 (62.45)	166,433 (76.14)	193,293 (74.77)	<0.0001
Several days	4081 (18.38)	3994 (22.50)	33,744 (15.44)	41,819 (16.18)	<0.0001
More than half the days	1422 (6.40)	1113 (6.27)	10,234(4.68)	12,769 (4.94)	<0.0001
Nearly everyday	905 (4.08)	1517 (8.54)	79,049 (3.62)	10,326 (3.99)	<0.0001
Feeling down, depressed, or hopeless					
Not at all	15,874 (71.49)	11,420 (64.37)	170,612 (78.06)	197,906 (76.56)	<0.0001
Several days	4451 (20.05)	4040 (22.77)	34,643(15.85)	43,134(16.690	<0.0001
More than half the days	1104 (4.97)	1038 (5.85)	7477 (3.42)	9619 (3.72)	<0.0001
Nearly everyday	774 (3.49)	1191 (6.71)	5679 (2.60)	7644 (2.96)	<0.0001
Trouble sleeping or sleeping too much					
Not at all	1211 (54.55)	8927 (50.39)	140,182 (64.14)	161,220 (62.37)	<0.0001
Several days	6080 (27.38)	4101(23.15)	48,611 (22.24)	58,792(22.75)	<0.0001
More than half the days	1734 (7.81)	1813 (10.23)	14,005 (6.41)	17,552(6.79)	<0.0001
Nearly everyday	2278 (10.26)	2848 (16.07)	15,672 (7.17)	20,798 (8.05)	<0.0001
Feeling tired or having little energy					
Not at all	8806 (39.66)	6603 (37.27)	111,048 (50.82)	126,457 (48.93)	<0.0001
Several days	8698 (39.17)	6067 (34.24)	74,239 (33.97)	89,004 (34.44)	<0.0001
More than half the days	2434 (10.96)	2013 (11.36)	17,045 (7.80)	21,492 (8.32)	<0.0001
Nearly everyday	2265 (10.20)	3011 (16.99)	16,109 (7.37)	21,385 (8.27)	<0.0001
Poor appetite or overeating					
Not at all	15,161 (68.28)	11,499 (64.90)	167,621 (76.70)	194,281 (75.17)	<0.0001
Several days	4414 (19.88)	3547 (20.02)	33,019 (15.11)	40,980 (15.86)	<0.0001
More than half the days	1432 (6.45)	1307 (7.38)	9881 (4.52)	12,620 (4.88)	<0.0001
Nearly everyday	1196 (5.39)	1364 (7.70)	7886 (3.61)	10,446 (4.04)	<0.0001
Feeling bad about yourself					
Not at all	17,685 (79.65)	13,599 (76.76)	184,941 (84.64)	2,162,259 (83.67)	<0.0001
Several days	3086 (13.90)	2443 (13.79)	23,477 (10.74)	29,006 (11.22)	<0.0001
More than half the days	847 (3.81)	655 (3.70)	5215 (2.39)	6717 (2.60)	<0.0001
Nearly everyday	585 (2.63)	1020 (5.76)	4709 (2.16)	6314 (2.44)	<0.0001
Trouble concentrating on things					
Not at all	17,690 (79.67)	13,468 (76.02)	184,226 (84.31)	215,384 (83.34)	<0.0001
Several days	2739 (12.34)	2553 (14.41)	21,977 (10.06)	27,269 (10.55)	<0.0001
More than half the days	850 (3.83)	814 (4.59)	6108 (2.80)	7772 (3.01)	<0.0001
Nearly everyday	924 (4.16)	861 (4.86)	6077 (2.78)	7862 (3.04)	<0.0001
Moving or speaking slowly or too fast					
Not at all	19,166 (86.32)	14,627 (82.56)	197,604 (90.43)	23,1397 (89.54)	<0.0001
Several days	1907 (8.59)	1722 (9.72)	13,309 (6.09)	16,938 (6.55)	<0.0001
More than half the days	619 (2.79)	649 (3.66)	4218 (1.93)	5486 (2.12)	<0.0001
Nearly everyday	494 (2.22)	719 (4.06)	3246 (1.49)	4459 (1.73)	<0.0001
Thought you would be better off dead					
Not at all	21,257 (95.74)	16,744 (94.51)	211,887 (96.98)	249,888 (96.70)	<0.0001
Several days	692 (3.12)	776 (4.38)	4254 (1.95)	5722 (2.21)	<0.0001
More than half the days	151 (0.68)	78 (0.44)	1287 (0.59)	1516 (0.59)	<0.0001
Nearly everyday	103 (0.46)	119 (0.67)	876 (0.40)	1098 (0.42)	<0.0001
Difficulty these problems have caused					
Not at all	12,392 (72.70)	9143 (65.07)	111,491 (76.84)	133,026 (75.50)	<0.0001
Several days	3692 (21.66)	3755 (26.73)	28,067 (19.34)	35,514 (20.16)	<0.0001
More than half the days	742 (4.35)	682 (4.85)	4013 (2.77)	5437 (3.09)	<0.0001
Nearly everyday	219 (1.28)	470 (3.35)	1427 (0.98)	2116 (1.20)	<0.0001

**Table 3:** Predictors of major depressive disorder and substance use in childhood and adult-onset asthma.

Childhood Asthma					Adult Asthma			
Effect	Adjusted Odds Ratio (aOR)	95% Wald Confidence Limits (95%CI)		*p* Values	Adjusted Odds Ratio (aOR)	95% Wald Confidence Limits (95%CI)		*p* Values
Major Depressive Disorder	0.38	0.38	0.38	<0.0001	2.64	2.64	2.64	<0.0001
High Alcohol Intake	1.06	1.06	1.06	<0.0001	0.95	0.94	0.95	<0.0001
Smoking	1.19	1.19	1.19	<0.0001	0.84	0.84	0.84	<0.0001
Marijuana	0.40	0.39	0.40	<0.0001	2.53	2.53	2.54	<0.0001
Cocaine	67.99	67.67	68.31	<0.0001	0.02	0.02	0.02	<0.0001
Heroin	0.55	0.55	0.55	<0.0001	1.82	1.82	1.83	<0.0001
Methamphetamine	1.67	1.67	1.67	<0.0001	0.60	0.60	0.60	<0.0001
Needle drug	0.71	0.71	0.71	<0.0001	1.41	1.41	1.41	<0.0001
c value	0.777				0.777			

## Data Availability

https://www.cdc.gov/nchs/nhanes/about_nhanes.htm (accessed on 11 April 2022).
